# Informant accuracy of IQCODE, AD8 and GPCOGi for diagnosis of dementia: does your friend know best?

**DOI:** 10.1186/s12875-025-02745-w

**Published:** 2025-04-15

**Authors:** Jasmine Chingono, Samuel Thomas Creavin, Mark Fish, Sarah Cullum, Antony Bayer, Sarah Purdy, Yoav Ben-Shlomo

**Affiliations:** 1 Bristol Medical School, University of Bristol, Bristol, UK; 2 Royal Devon and Exeter NHS Foundation Trust, Exeter, UK; 3 Department of Psychological Medicine, School of Medicine, The University of Auckland, Grafton, New Zealand; 4 School of Medicine, Cardiff University, Cardiff, UK

**Keywords:** Dementia, Neurocognitive disorders, Informant, Relationship, Accuracy, IQCODE, AD8, GPCOG, Primary care

## Abstract

**Background:**

Increasing numbers of people require evaluation for possible dementia. However, research on the accuracy of informant questionnaires in primary care remains limited.

**Methods:**

This study assessed the diagnostic accuracy of IQCODE, AD8, and GPCOGi based on the informant’s relationship to the patient. We recruited 240 participants from 21 general practices in South West England. The reference standard for a diagnosis of dementia was made by a specialist clinician using ICD-10 criteria. A threshold of greater than 3.3 on IQCODE, greater or equal to 2 on AD8 and less than 5 on the informant component of GPCOG was used to indicate an abnormal test.

**Results:**

Of 238 participants with informant data, 131 had dementia, 60 had CIND, and 47 had normal cognition. Median informant age was 70 years (IQR 60 years to 78 years). 71% of informants were female and 56% were spouses. On all three questionnaires, compared to spouses, adult descendants tended to score participants more cognitively impaired, whereas friends scored participants less cognitively impaired. However, there was little evidence of difference by informant type once fully adjusted. Sensitivity by informant type ranged from 91 to 100% for IQCODE, 94–100% for AD8 and 99% to100% for GPCOGi. There was no significant difference in sensitivity by informant type. Specificity by informant type ranged from 25 to 79% for IQCODE, 13–75% for AD8 and 17–38% for GPCOGi. Adult descendants tended to have the lowest specificity at 25% (95% CI 10–47%) for IQCODE, 13% (95% CI 3–32%) for AD8 and 17% (95% CI 5–37%) for GPCOGi. Friends tended to have the highest specificity at 79% (95% CI 49–95%) for IQCODE, 75% (95% CI 48–93%) for AD8 and 38% (95% CI 15–64%) for GPCOGi.

**Conclusions:**

An informant of any relationship type, using IQCODE, AD8 or GPCOGi may be useful for ruling out dementia but not for ruling it in. We found no evidence of difference between spouse or adult descendants but friends performed significantly better overall on IQCODE and AD8.

## Introduction

Dementia is a syndrome of progressive, cognitive symptoms including memory disturbance, difficulties with language, executive function, visuospatial skills and changes in behaviour [1]. International Classification of Disease 11 (ICD-11) and Diagnostic and Statistical Manual of Mental Disorders 5 (DSM-5) now use the terminology neurocognitive disorders (NCDs) [2, 3]. This marks a shift from previous terminology used in DSM-IV and ICD-10 and it is hoped that in time NCDs will become the common language for researchers, clinicians and the public [4, 5]. However, to align with the terminology most commonly used in England, the origin of the study, the term “dementia” is utilised [1].

Timely and accurate diagnosis allows patients and their carers to access appropriate medication, services and engage in advanced care planning [6]. However, the path to a dementia diagnosis can be lengthy, with patients and their carers experiencing considerable uncertainty and a lack of patient-centred support [7].

Many people living with cognitive problems do not have a formal diagnosis of dementia, and pressure on diagnostic pathways is anticipated to increase in future [8, 9]. In the United Kingdom, a dementia diagnosis can require referral to a specialist dementia diagnostic service, and the role of primary care in supporting a more effective pathway to diagnosis is a priority research area [10, 11].

An informant is a person close to a patient, such as a family member, friend, or caregiver, who can provide additional information to aid diagnosis. The use of a structured informant questionnaire is advocated by the National Institute for Health and Care Excellence (NICE) as part of an initial assessment [10]. Informant questionnaires can be used as part of a wider assessment to support a diagnosis of dementia but in isolation are not diagnostic. There are number of informant questionnaires available but not all have validated for use in primary care settings [12]. This study focuses on the three informant questionnaire which are considered to have been appropriately validated for use in primary care: Informant Questionnaire on Cognitive Decline in the Elderly (IQCODE), Ascertain Dementia 8 (AD8) and General Practitioner Assessment of Cognition (GPCOG) [13–15]. GPCOG, consists of two parts and includes a component for the informant to complete (GPCOGi) [15]. All three informant questionnaires ask the informant to rate the patient’s performance on several cognitive indicators compared to several years ago.

Although informant questionnaires are often used in conjunction with other tools to aid assessment of cognition, there is currently no agreed gold standard [12]. A recent overview of informant questionnaires for dementia highlighted that whilst IQCODE and AD8 have the greatest evidence base, there is a lack of diagnostic accuracy evaluations in primary care and insufficient evidence to compare the questionnaire performance. Many informant questionnaires had only been evaluated in a limited number of studies and many lacked either an evaluation of bias or were deemed to be at risk of bias. The test accuracy of IQCODE and AD8 had been most widely evaluated. However, the study authors GRADE rated the evidence as low due to risk of bias in the included studies and imprecision around pooled sensitivity and specificity. In addition, the majority of studies were within secondary care and a there was a lack of diagnostic accuracy evaluations in primary care [16].

Informant characteristics may affect scoring and therefore accuracy of informant tests. For example, a study found that wives with a higher level of anxiety or depressive symptoms tended to rate more cognitive impairment in their husbands on the IQCODE [17]. However, previous studies suggest that IQCODE scores are not influenced by length or type of relationship, age, or educational status of the informant [17, 18]. These studies examined associations between tests scores and relationship types, rather than test accuracy, which is more clinically relevant.

The aim of this study was therefore to address these research gaps by exploring the diagnostic accuracy of IQCODE, AD8 and GPCOGi in a primary care setting, and whether this differed by informant characteristics, particularly relationship type.

## Method

This was a prospective diagnostic accuracy study.

### Population

We recruited participants between March 2015 and May 2017 from 21 general practitioner (GP) surgeries in the Bristol, North Somerset and South Gloucestershire (BNSSG) area in the South West of England. This covered a diverse area around the city of Bristol, with a total population of around 900,000, with 12% aged over 70 [19].

### Inclusion and exclusion criteria

We included people aged over 70 years who were seeking evaluation for concerns about cognition but did not have an existing formal diagnosis of dementia. We did not specify the nature or severity of the cognitive symptoms. However, these symptoms must have been present for at least six months and reported by the person themselves, someone close to them, or a healthcare professional. We required that an informant attended the research clinic as a criterion for participation, to enable robust diagnosis.

We excluded people with a known neurological disorder (including Parkinsonism, multiple sclerosis, learning disability, and Huntington’s disease), a psychiatric disorder requiring secondary care input, those registered blind, profound deafness preventing the use of a telephone, those with rapidly progressive symptoms and those with advanced cognitive problems unable to consent.

The person accompanying the participant had to be willing to act as an informant and complete the informant questionnaires. There were no additional inclusion or exclusion criteria for informants. However, it was encouraged that they had known the participant for at least 10 years.

We encouraged GPs to refer all consecutive eligible patients regardless of their clinical judgement or any initial test results.

Those referred to the study by their GP were contacted by the team to confirm eligibility and provided further written details of the study. Written informed consent was required by both the participant and the informant. Further details have been reported previously [20].

### Index test

We asked informants to complete the IQCODE-16, GPCOGi, and AD8 during the research clinic visit facilitated by a single GP. All three questionnaires have previously been published elsewhere and were performed as outlined by the original authors [13–15]. The facilitating GP was not aware of any other clinical information related to the participant or informant.

The IQCODE-16 asks the informant to rate the patient from 1: “much improved” to 5: “much worse” now, compared to 10 years ago, across 16 questions and a mean score calculated. The original version of IQCODE consisted of 26 items. Internal consistency is high with Cronbach’s alpha between 0.93 and 0.97 [21]. A short form, consisting of 16 items was subsequently developed and found to have comparable validity [13].

AD8 consists of eight questions assessing whether for each statement there has been a change in the last several years caused by cognitive problems. Response options are: “yes, a change”, “no, no change” or “N/A, don’t know”. Answers “yes, a change” score 1 and other responses do not score. The AD8 can be completed by a patient or informant [14]. AD8 performs well on internal consistency with Cronbach’s alpha 0.84 [22].

Unlike IQCODE and AD8, GPCOG includes both a patient and informant component. The informant component consists of six questions asking the informant to compare the patient to how they were compared to 5 to 10 years ago. Response options are “yes”, “no”, “unsure”, “N/A”. All responses except “yes” score 1 point. Only the informant scores were used for analysis in this study. We refer to this as GPCOGi. GPCOGi performs well on internal consistency with Cronbach’s alpha 0.80 [15].

We used a threshold of > 3.3 as a threshold for an abnormal index test on IQCODE (mean scores range 1–5; higher scores indicating worse cognition). This reflected the threshold used for the pooled analysis in the Cochrane systematic review [16].

We used a threshold of greater or equal to 2 on AD8 (scores range 0–8; higher scores indicating worse cognition). This reflected the threshold defined by the original author [14].

We used a threshold < 5 on the GPCOGi (max score 6, lower scores indicating worse cognition). This reflected the reported threshold for the informant component of the GPCOG by the original author [15].

We categorised informant relationship types as spouse, adult descendant, sibling or friend, and collected information on informant age and sex, all based on self-report.

### Reference standard

A single specialist physician assessed participants over approximately one hour using a clinical history, Addenbrooke’s Cognitive Examination III (ACE-III), Brief Assessment Schedule Depression Cards (BASDEC) and the informant-completed Bristol Activities of Daily Living (BADL) Questionnaire [23–25]. The specialist physician did not use specific thresholds for these measures and was not aware of any GP judgement, other test results or investigations. The specialist used their integrated assessment to consider a dementia diagnosis according to ICD-10 criteria [4]. The specialist physician assigned a diagnosis of Cognitive Impairment Not Dementia (CIND) if their assessment met the criteria for Petersen mild cognitive impairment, or another cause of cognitive impairment such as traumatic brain injury or affective disorder [26]. We reviewed medical records for all patient participants six months after the research clinic to find subsequent information that would contradict the initial research clinic judgement. For borderline cases a second independent specialist reviewed initial assessment as well as the medical record to determine a final diagnostic classification.

### Statistical methods

We summarised information on participants and the variation across cognitive categories and informant types. We used linear regression to test the hypothesis of no association between continuous informant test score and categorical informant type (adult descendant, sibling, friend), compared to spouse as the baseline category. We derived diagnostic accuracy parameters with 95% confidence intervals for informant tests at pre-specified thresholds (see Index Tests) compared to reference standard dementia as determined by specialist assessment. We calculated sensitivity and specificity of informant tests at these thresholds in subgroups to derive accuracy by informant type and sex. We used logistic regression and t tests to investigate potential associations between a missing informant score and informant relationship type, age, and sex. We used Stata 15 for analysis.

## Results

### Participants

**Fig. 1:**
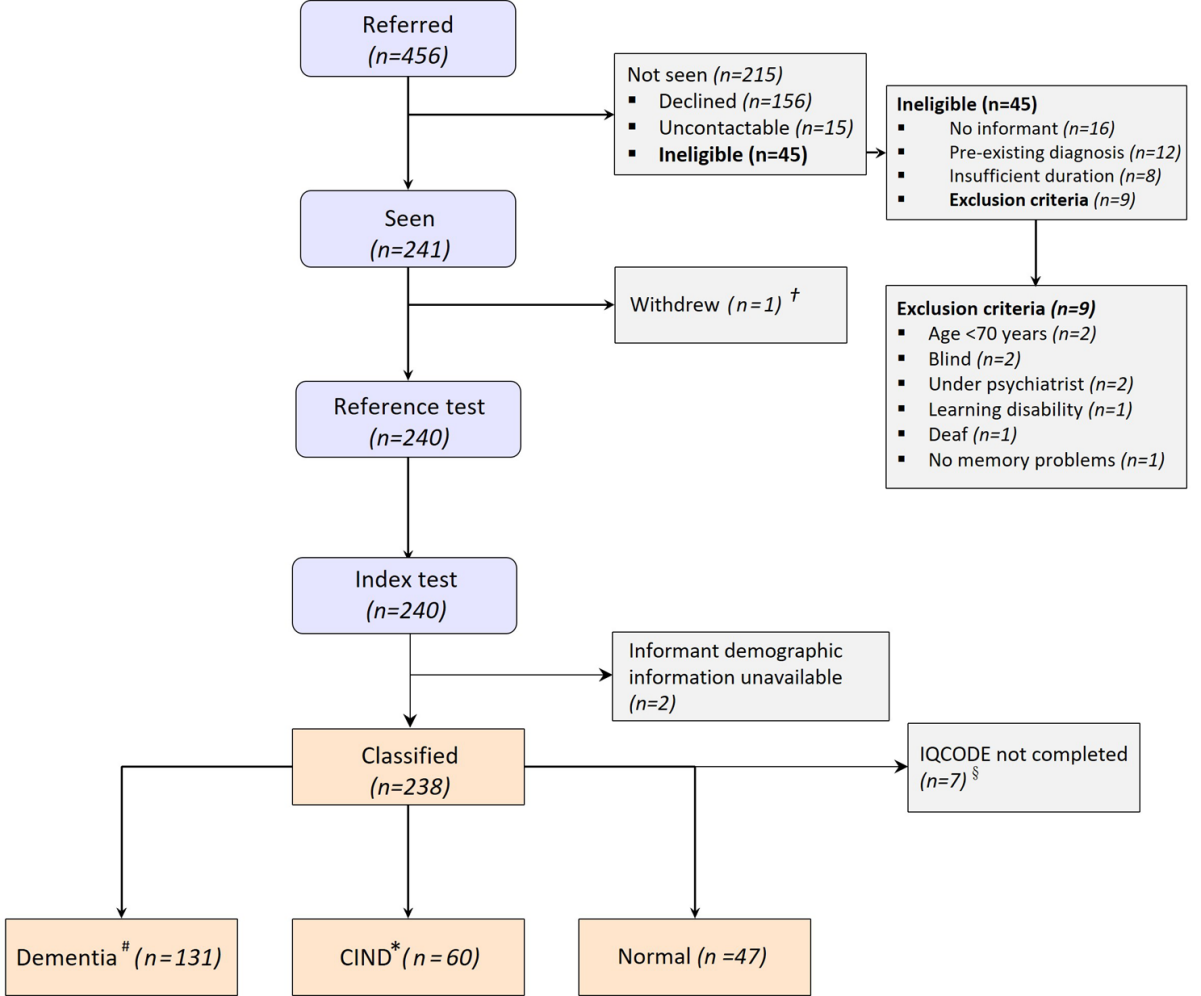
STARDem flowchart for study inclusion of participants. ^†^One person withdrew as acutely unwell. ^§^IQCODE was offered to all informants. Seven declined. ^#^ Dementia according to International Classification of Disease 10th Revision (ICD-10) definition. ^*^Cognitive impairment not including dementia (CIND) defined as Petersen mild cognitive impairment or another cause of cognitive impairment such as traumatic brain injury or affective disorder

We recruited 240 participants. Figure 1 shows a flowchart of study inclusion. We had demographic information available for 238 informants, and of these 231 completed IQCODE, and 238 completed AD8 and GPCOGi. The specialist classified all 240 patient participants according to the reference standard: ‘dementia’ (*n* = 132), ‘CIND’ (*n* = 61) and ‘normal’ cognition (*n* = 47). For the 238 who also had informant information, the specialist classified the patient-participants as: ‘dementia’ (*n* = 131), ‘CIND’ (*n* = 60) and ‘normal’ cognition (*n* = 47).

Table 1 summarises informant characteristics and informant questionnaire score by participant cognitive category according to the reference standard.

The median informant age was 70 years (IQR 60 years to 78 years). Informants for people classified as having dementia tended to be younger (median age 69 years) than informants for people classified as having normal cognition (median age 71 years) or has having CIND (median age 73 years). Most informants were women (71%), and this was similar for patients categorised as having dementia, CIND or being cognitively normal.

The most common informant type overall was spouse, 56% (*n* = 134). People classified as having dementia were more often accompanied by an adult descendant (43%) than people classified as having CIND (28%) or normal cognition (15%), OR 4.3 (95% CI 1.7 to 11; *p* = 0.0006).

**Table 1:** Characteristics of informants by patient participant cognitive category

Informant Characteristic	Cognitive category		
	Dementia^a^ (*n* = 131)	CIND^b^ (*n* = 60)	Normal cognition (*n* = 47)
Median age of informant at clinic, years (IQR)	69 (59–78)	73 (58–77)	71 (65–77)
Sex (column %)^c^			
Male *n* = 62 (26)	37 (28)	13 (22)	12 (26)
Female *n* = 169 (71)	93 (71)	46 (77)	30 (64)
Informant type, n (column %)			
Spouse *n* = 134 (56)	69 (53)	36 (60)	29 (62)
Adult descendant *n* = 80 (34)	56 (43)	17 (28)	7 (15)
Sibling *n* = 4 (2)	2 (2)	2 (3)	0 (0)
Friend *n* = 20 (8)	4 (3)	5 (8)	11 (23)
**IQCODE**			
Mean IQCODE median score^d^ (IQR)	4.3 (3.8–4.6)	3.5 (3.3–4.1)	3.25 (3.1 to 3.4)
*People in each cognitive category whose score exceeded the abnormal IQCODE threshold*,* for each informant type*,* n (%)*^*e*^			
Spouse (total *n* = 129)	63 (91)	25 (78)	15 (54)
Adult descendant (total *n* = 80)	56 (100)	13 (76)	5 (71)
Sibling (total *n* = 4)	2 (100)	1 (50)	0 (0)
Friend (total *n* = 18)	4 (100)	2 (50)	1 (10)
**AD8**			
AD8 median score^f^ (IQR)	7 (5–8)	4 (2–6)	2 (0–3)
*People in each cognitive category whose score exceeded the abnormal AD8 threshold*,* for each informant type*,* n (%)*^*g*^			
Spouse (total *n* = 134)	65 (94)	26 (72)	21 (72)
Adult descendant (total *n* = 80)	56 (100)	16 (94)	5 (71)
Sibling (total *n* = 4)	2 (100)	1 (50)	-
Friend (total *n* = 20)	4 (100)	2 (40)	2 (18)
**GPCOGi**			
GPCOGi total median score^h^ (IQR)	1 (1–3)	3 (2–4)	4 (3–5)
*People in each cognitive category whose score exceeded the abnormal GPCOGi threshold*,* for each informant type*,* n (%)*^*i*^			
Spouse (total *n* = 134)	68 (99)	32 (89)	22 (76)
Adult descendant (total *n* = 80)	56 (100)	15 (88)	5 (71)
Sibling (total *n* = 4)	2 (100)	2 (100)	-
Friend (total *n* = 20)	4 (100)	5 (100)	5 (45)

^a^ Dementia according to International Classification of Disease 10th Revision (ICD-10) definition. ^b^Cognitive impairment not including dementia (CIND) defined as Petersen mild cognitive impairment or another cause of cognitive impairment such as traumatic brain injury or affective disorder. ^c^ Sex was not recorded for 7 participants. ^d^ Mean IQCODE scores range 1 to 5, higher scores indicating worse cognition ^e^ IQCODE threshold of mean score > 3.3 indicates abnormal test. For example, of the 131 people classified as having dementia, there were 69 who were rated by a spouse informant, and of these 63 (91%) exceeded the abnormal threshold on IQCODE. ^f^AD8 scores range from 0 to 8, higher scores indicate worse cognition. ^g^AD8 threshold of ≥2 indicates abnormal test. ^h^GPCOGi scores range from 0 to 6, lower scores indicate worse cognition. ^i^GPCOGi threshold of < 5 indicates abnormal test

Table 1 also shows that people classified as having dementia had higher mean IQCODE and total AD8 scores (median 4.3 and 7 respectively) than people classified as having CIND (median 3.5 and 4 respectively) and normal cognition (median 3.25 and 2 respectively). People classified as having dementia had lower total scores on GPCOGi (median 1) than people classified as having CIND (median 3) and normal cognition (median 4).

### Informant questionnaire scores by informant characteristics

Adult descendants tended to score participants *more* cognitively impaired on all three informant questionnaires when compared to spouses as the baseline informant type. However, there was no difference after adjustment for informant age, participant age, informant sex and diagnosis. Friends tended to score participants *less* cognitively impaired on all three informant questionnaires when compared to spouses. Once adjusted for informant age, participant age, informant sex and diagnosis, this difference attenuated for AD8 but remained statistically significant (-1.55, 95% CI -2.74 to -0.36. *p* = 0.01). However, there was no difference for IQCODE and GPCOGi. Siblings did not score participants significantly different on all three informant questionnaires when compared to spouses. (Tables 2 and 3).

There was no association between informant sex and IQCODE, AD8 or GPCOGi score.

With each additional five years in informant age, informants tended to score participants less cognitively impaired on IQCODE and AD8. However, once adjusted for participant age, informant sex and diagnosis, this difference attenuated for IQCODE and there was no difference for AD8. (Tables 2 and 3)

**Table 2:** Informant questionnaire scores by informant characteristics, unadjusted

Informant Relationship Type^a^				
	Spouse	**Adult descendant**	**Sibling**	**Friend**
IQCODE	-	0.42 (0.25, 0.59)	-0.18 (-0.78, 0.42)	-0.55 (-0.85, -0.25)
AD8	-	1.73 (1.04, 2.43)	0.52 (-1.97, 3.02)	-2.48 (-3.66, -1.30)
GPCOGi	-	-0.63 (-1.06, -0.20)	-0.24 (-1.80, 1.32)	0.81 (0.07, 1.54)
**Informant Sex** ^**b**^				
	**Female**		**Male**	
IQCODE	-		0.09 (-0.10, 0.29)	
AD8	-		-0.03 (-0.82, 0.77)	
GPCOGi	-		-0.15 (-0.61, 0.30)	
**Informant Age** ^**c**^				
Difference in score for every additional five years in informant age				
IQCODE	-0.06 (-0.09, − 0.03)			
AD8	-0.25 (-0.385, -0.114)			
GPCOGi	0.04 (-0.04, 0.12)			

^a^ Difference in score when compared to spouses as the baseline informant type. ^b^ Difference in score when compared to male as the baseline informant sex. ^c^ Difference in score when compared to normal cognition as the baseline diagnosis

**Table 3:** Informant questionnaire scores by informant characteristics, adjusted

Informant Relationship Type^a^						
	Spouse		Adult descendant	Sibling		Friend
IQCODE	-		-0.07 (-0.42, 0.28)	-0.35 (-0.88, 0.17)		-0.26 (-0.57, 0.05)
AD8	-		0.38 (-1.02, 1.78)	0.05 (-2.10, 2.20)		-1.55 (-2.74, -0.36)
GPCOGi	-		0.03 (-0.85, 0.91)	0.09 (-1.26, 1.43)		0.06 (-0.69, 0.80)
**Informant Sex** ^**b**^						
	**Female**			**Male**		
IQCODE	-			0.08 (-0.08, 0.24)		
AD8	-			-0.12 (-0.76, 0.53)		
GPCOGi	-			-0.10 (-0.50, 0.30)		
**Informant Age**						
	Difference in score for every additional five years in informant age					
IQCODE	-0.06 (-0.12, -0.003)					
AD8	-0.15 (-0.39, 0.10)					
GPCOGi	0.01 (-0.14, 0.17)					
**Participant Age**						
Difference in score for every additional five years in participant age						
IQCODE	0.09 (0.01, 0.17)					
AD8	0.12 (-0.22, 0.45)					
GPCOGi	-0.22 (-0.43, -0.01)					
**Diagnosis** ^**c**^						
	**Normal cognition**	**CIND**			**Dementia**	
IQCODE	-	0.31 (0.1, 0.53)			0.76 (0.56, 0.96)	
AD8	-	1.11 (0.25, 1.98)			3.36 (2.55, 4.16)	
GPCOGi	-	-0.78 (-1.32, -0.24)			-1.84 (-2.34, -1.34)	

Adjusted for informant type, informant sex, informant age, participant age and diagnosis

^a^ Difference in score when compared to spouses as the baseline informant type. ^b^ Difference in score when compared to male as the baseline informant sex. ^c^ Difference in score when compared to normal cognition as the baseline diagnosis

### Diagnostic accuracy

The diagnostic accuracy of IQCODE, AD8 and GPCOGi are summarised in Tables 4, 5 and 6 respectively.

**Table 4:** Accuracy of informant judgement for the diagnosis of dementia using IQCODE

	AUROC (95 CI %)	Sensitivity (95 CI %)	Specificity (95 CI %)	PPV (95 CI %)	NPV (95 CI %)
Overall	0.67 (0.62, 0.72)	95 (90, 98)	38 (29, 48)	67 (60, 74)	86 (73, 95)
**Informant Relationship Type**					
Spouse	0.62 (0.55, 0.69)	91 (82, 97)	33 (22, 47)	61 (51, 71)	77 (56, 91)
Adult descendant	0.63 (0.54, 0.71)	100 (94, 100)	25 (10,47)	76 (64, 85)	100 (54, 100)
Sibling	0.75 (0.26, 1)	100 (16, 100)	50 (1, 99)	67 (9, 99)	100 (3, 100)
Friend	0.89 (0.78, 1)	100 (40, 100)	79 (49, 95)	57 (18, 90)	100 (72, 100)
**Informant Sex**					
Male informant	0.74 (0.63, 0.85)	97 (86, 100)	50 (28, 72)	77 (62, 88)	92 (62, 100)
Female informant	0.63 (0.57, 0.69)	95 (88, 98)	32 (21, 44)	64 (56, 72)	82 (63, 94)

AUROC = areas under the receiver operating characteristic curve. PPV = positive predictive value. NPV = negative predictive value. 95% CI = 95% Confidence interval

**Table 5:** Accuracy of informant judgement for the diagnosis of dementia using AD8

	AUROC (95 CI %)	Sensitivity (95 CI %)	Specificity (95 CI %)	PPV (95 CI %)	NPV (95 CI %)
Overall	0.64 (0.60, 0.69)	97 (92, 99)	32 (23, 42)	64 (56, 70)	90 (75, 97)
**Informant Relationship Type**					
Spouse	0.61 (0.55, 0.67)	94 (86, 98)	28 (17, 40)	58 (48, 67)	82 (60, 95)
Adult descendant	0.56 (0.50, 0.63)	100 (94, 100)	13 (3,32)	73 (61, 82)	100 (30, 100)
Sibling	0.7 (0.26, 1)	100 (16, 100)	50 (1, 99)	67 (9, 99)	100 (3, 100)
Friend	0.88 (0.77, 0.99)	100 (40, 100)	75 (48, 93)	50 (16, 84)	100 (74, 100)
**Informant Sex**					
Male informant	0.72 (0.62, 0.82)	100 (91, 100)	44 (24, 65)	73 (58, 84)	100 (72, 100)
Female informant	0.60 (0.55, 0.66)	96 (89, 99)	25 (16, 36)	61 (53, 69)	83 (61, 95)

AUROC = areas under the receiver operating characteristic curve. PPV = positive predictive value. NPV = negative predictive value 95%. CI 95%. Confidence interval

**Table 6:** Accuracy of informant judgement for the diagnosis of dementia using GPCOGi

	AUROC (95 CI %)	Sensitivity (95 CI %)	Specificity (95 CI %)	PPV (95 CI %)	NPV (95 CI %)
Overall	0.59 (0.56, 0.63)	99 (96, 100)	20 (13, 28)	60 (53, 67)	96 (77, 100)
**Informant Relationship Type**					
Spouse	0.58 (0.53, 0.63)	99 (92, 100)	17 (9, 28)	56 (47, 65)	92 (62, 100)
Adult descendant	0.58 (0.51, 0.66)	100 (94, 100)	17 (5, 37)	74 (62, 83)	100 (40, 100)
Sibling^a^	-	-	-	-	-
Friend	0.69 (0.57, 0.81)	100 (40, 100)	38 (15, 64)	29 (8, 58)	100 (54, 100)
**Informant Sex**					
Male informant	0.62 (0.54, 0.71)	100 (91, 100)	24 (9, 45)	66 (52, 54)	100 (54, 100)
Female informant	0.57 (0.53, 0.62)	99 (94, 100)	16 (8, 26)	59 (51, 67)	92 (64, 100)

AUROC = areas under the receiver operating characteristic curve. PPV = positive predictive value. NPV = negative predictive value 95%. CI 95% = Confidence interval

^a^All four informants classed as siblings categorised patient participants as impaired on GPCOGi

#### All informants

Overall sensitivity (that is, ability to detect correctly someone with dementia) was 95% (95% CI 90–98%) for IQCODE, 97% (95% CI 92–99%) for AD8 and 99% (95% CI 96–100%) for GCPOGi. Overall specificity (that is, ability to detect correctly someone without dementia) was 38% (95% CI 29–48%) for IQCODE, 32% (95% CI 23–42%) for AD8 and 20% (95% CI 13–28%) for GPCOGi.

Overall accuracy was similar for all informant questionnaires with area under the receiver operating characteristic curve (AUROC) of 0.67 (95% CI, 0.62 to 0.72) for IQCODE, 0.64 (95% CI, 0.60 to 0.69) for AD8 and 0.59 (95% CI, 0.56 to 0.63) for GPCOGi, though the 95% confidence intervals only just overlapped for IQCODE and GPCOGi.

#### Informants by relationship type

Sensitivity was consistently high across all informant relationship types for all three informant questionnaires. Sensitivity by informant relationship type ranged from 91 to 100% for IQCODE, 94–100% for AD8 and 99% to100% for GPCOGI. Spouses tended to have the lowest sensitivity. However, sensitivity CIs overlap across all informant types on IQCODE, AD8 and GPCOGi and these differences were consistent with chance.

Specificity was lower across all informant relationship types for all three informant questionnaires. Specificity by informant relationship type ranged from 25 to 79% for IQCODE, 13–75% for AD8 and 17–38% for GPCOGi. Adult descendants tended to have the lowest specificity at 25% (95% CI 10–47%) for IQCODE, 13% (95% CI 3–32%) for AD8 and 17% (95% CI 5–37%) for GPCOGi. Friends tended to have the highest specificity at 79% (95% CI 49–95%) for IQCODE, 75% (95% CI 48–93%) for AD8 and 38% (95% CI 15–64%) for GPCOGi.

AUROC by informant relationship type ranged from 0.62 to 0.89 for IQCODE, 0.56 to 0.88 for AD8 and 0.58 to 0.69 for GPCOGi. Friends tended to have the highest AUROC at 0.89 (95% CI 0.78 to 1) for IQCODE, 0.88 (95% CI 0.77 to 0.99) for AD8 and 0.69 (95% CI 0.57 to 0.81) for GPCOGi. It was not possible to calculate a p value for GPCOGi because there were zero siblings who rated their participant as being cognitively healthy. However, there was strong evidence against the null hypothesis of no difference by informant type for IQCODE (*p* = 0.0004) and AD8 (*p* = 0.0001), suggesting that overall friends are more accurate when using IQCODE and AD8, due to an increase in the specificity, without loss of sensitivity.

There was substantial uncertainty in the estimates for friends and siblings with large confidence intervals, as there were small numbers of these informants.

#### Informants by sex

Males had a higher AUROC for all three tests, driven by higher specificity for males. However, 95% CIs overlapped and were consistent with chance.

#### Missing IQCODE scores

We found no association between informant sex and missing IQCODE. The odds ratio for not completing IQCODE was 2.1 (95% 0.46 to 9.6, *p* = 0.35) when male informants were compared to female informants.

We did find an association between missing IQCODE and informant age. The seven informants who did not complete IQCODE were on average 12 years older (95% CI 2 years to 21 years) than those who did complete the IQCODE; t test *p* = 0.02.

## Discussion

This is the first study to our knowledge to explore diagnostic accuracy by informant relationship type. Overall, all three informant tests had relatively high sensitivity, low specificity and had a similar AUROC. Compared to spouses, adult descendants tended to score participants more cognitively impaired, whereas friends scored participants less cognitively impaired on all three questionnaires. However, there was little evidence of scoring difference by informant relationship type once fully adjusted, except for friends who scored participants lower on AD8. For diagnostic accuracy, we found little difference between spouse and adult descendants, who made around 90% of informants. In contrast only 8% of informants were a friend, but they performed significantly better overall on IQCODE and AD8 due to an increase in specificity without loss of sensitivity. Clinicians have little control over who accompanies a patient to an appointment or is available to complete an informant questionnaire. The sample size for friends was small. However, this finding may challenge assumptions that relatives may know best. Pragmatically, our consistently high reported sensitivity across informant types suggests an informant of any relationship type may be useful at ruling out dementia.

## Comparison with existing literature

Although common thresholds for detecting dementia using IQCODE tend to be applied (often ranging from 3.3 to 3.6), there is no universally accepted threshold, making comparison across studies challenging [27]. Previous literature, which considers studies with varying thresholds, suggests a pooled estimate of IQCODE sensitivity of 80–91% and specificity of 65–85% but study quality is noted to be poor [16]. A systematic review found only one previous suitable study conducted in primary care [28]. The identified study had a sensitivity of 100% and specificity of 82% using a threshold of 3.3. The study reported a decreasing sensitivity and increasing specificity as the threshold increased and suggested an optimal cut-point of 3.35 using AUROC analyses [29]. We found a similarly high sensitivity but a notably lower specificity in comparison to that study which may reflect aspects of the study design. For example, the other study conducted IQCODE over the telephone, had a lower prevalence of dementia (7% compared to 54% in our study), had a similar proportion of informants that were a spouse (47%), and was assessed as being at high risk of bias in all four Quality Assessment of Diagnostic Accuracy Studies (QUADAS) domains [28, 29].

The original author for AD8 defined a threshold of 2 [14]. However, systematic reviews identified that thresholds applied in studies have differed and result in differing test performance [30, 31]. Only one study in a primary care setting was identified. It reported a sensitivity of 90% and specificity of 88% at a threshold of ≥ 3 [32]. This limits the comparability to our reported sensitivity of 97% and specificity of 32% at a threshold of ≥ 2. However, it is perhaps worth noting that pooled analysis across healthcare settings found a sensitivity of 92% and specificity of 64% at a threshold of ≥ 2 compared to a sensitivity of 91% and specificity of 76% at a threshold of ≥ 3 [31]. The only study in primary care was assessed as being at risk of bias in one QUADAS domain and unclear in two [31].

There is less data available to evaluate the use of GPCOG. The original study reported a sensitivity of 85% and specificity of 86% when used as the full two-stage questionnaire. The informant component used in isolation had a reported sensitivity of 89% and specificity of 66% [15]. We found higher sensitivity, 99%, and a notably lower specificity, 20%, in comparison to the original study which may reflect aspects of the study design. We only included those over age 70 where a concern about cognition had been raised and there was no formal diagnosis of dementia. However, the original GPCOG study included all adults over 75 regardless of cognitive status and those aged 50 to 74 with suspected cognitive impairment [15].

All informants completed AD8 and GPCOG. This is consistent with their reported ease of administration including fast completion and minimal training requirements [15, 30]. Not all informants completed IQCODE in this study, but completion was still high at 97%. An in-hospital study found poor uptake and completion rate of IQCODE by informants. It is likely that this reflected the inpatient nature of the study, and the authors suggested that short admissions and relatives being away from the ward during working hours may have been contributing factors [33]. Conversely, a community study suggested IQCODE was acceptable to informants and easy to use, which is in keeping with our findings [34].

Informant characteristics may influence test scores. However, reported data on the influence of informant characteristics is limited and variable. One study reported that the age of informants who were wives was not associated with IQCODE scoring [17, 18]. When considering all informant types, we found older informants scored participants less cognitively impaired on IQCODE. We found no association between AD8 or GPCOGi score and informant age when adjusted for informant sex and informant type.

Some investigators have reported that IQCODE is not influenced by relationship type or length [18], whereas others have found that spouses reported better cognitive function for patients than non-spouses [35]. We found that friends, compared to spouses, tended to score participants less cognitively impaired on IQCODE and also AD8.

## Strengths and limitations

This study used ICD-10 criteria as the reference standard for diagnosis. Since the completion of data collection in 2017, ICD-11 was published in 2018 and came into effect in 2022 [3]. ICD-11 reclassifies syndromes of impaired cognition that were under organic mental health conditions in ICD-10 to neurocognitive disorders. In doing so, ICD-11 now more closely reflects the change in terminology from DSM-IV to DSM-5 [2, 5]. This study’s use of CIND is perhaps most synonymous mild neurocognitive disorders in ICD-11. However, we have retained the terminology used in ICD-10 as this was the active criteria at the time of data collection and as is therefore the correct representation of diagnostic standard applied. We recognise that the now outdated use of ICD-10 perhaps limits generalisability of the findings.

We recruited participants from several GP surgeries, across a geographically diverse area. Cognitive symptoms could be identified by the person themselves, someone close to them or a healthcare professional but this information was not collected. The recruitment of participants via routine consultations where subjective cognitive symptoms were identified may be considered a strength as it is reflective of real world clinical practice. Although we made efforts to maximise inclusion, including providing translation services, participants were mostly white, native English speakers. IQCODE is, however, thought to be relatively unaffected by language and AD8 has been translated and validated in a variety of languages [27, 31]. To further determine whether our findings are generalise across a wider population, future may research may wish consider additional factors such as deprivation and ethnicity.

Recruitment to this study primarily focused on participant eligibility with a pragmatic approach to informant inclusion. No specific inclusion or exclusion criteria were applied to informants. It was encouraged that informants had known the participant for at least 10 years but this was not a requirement. It was assumed that an accompanying informant had sufficient knowledge of the participant to complete the informant questionnaire, as is often the case in clinical practice.

In this study we focus on relationship type as the proxy for familiarity. However, familiarity may be influenced a number of factors such as the duration of the relationship, frequency of contact and involvement before and during symptoms development. This data was not collected and therefore couldn’t be consider in the analysis.

This study did adjust for informant characteristics such as gender and age. However, informants’ own cognitive status was not measured. This reflects the reality of clinical practice, where it often assumed that the informant’s cognition is sufficient to complete the informant questionnaire. However, we recognise that this is a limitation of the study. It is likely some informants, particularly spouses, may have been cognitively impaired and this could have influenced their perception of the participants cognition. Determining the cognitive status of informants poses ethical challenges of assessing those not presenting with cognitive symptoms and may have acted as a barrier to participation.

This study has limited applicability to people with advanced cognitive impairment, a known neurological disorder or significant psychiatric commodities, since we excluded those people.

The uncertainty in the estimates increases when analysing by informant subtype, which limits the extent to which we can draw comparisons between categories; the number of informants in the sibling category was especially small, with large confidence intervals. Whilst we would expect informants attending the research clinic to mirror similar patterns to those who may routinely accompany patients to appointments, we could not investigate this formally. Index test informant measures (IQCODE, AD8, GPCOGi) were not used by the expert assessors when deciding the reference standard diagnosis, but the same informant who completed these index tests contributed to the expert evaluation and completed BADL, though this formed only one part of the holistic expert judgement about the diagnosis.

We applied a commonly used threshold for IQCODE and AD8, but there are a range of thresholds [27, 30, 31]. The accuracy at other thresholds may be different and was not explored in this analysis.

GPCOG consists of two components, a patient and informant questionnaire but we evaluated the informant component of GPCOG in isolation because we were interested specifically in informant characteristics [15].

## Conclusion

Overall, informants, when using IQCODE, AD8 or GPCOGi perform well on sensitivity but are less specific. We found little difference between spouse and adult descendants, who made around 90% of informants. In contrast only 8% of informants were a friend, but they performed significantly better overall on IQCODE and AD8 due to an increase in specificity without loss of sensitivity. In clinical practice, there is little control over who may accompany a patient to appointments or be available to complete an informant questionnaire. However, given our findings show consistently high sensitivity but variable specificity, we suggest that informants, of any relationship type, when using IQCODE, AD8 or GPCOGi may be useful for ruling out dementia but not for ruling it in.

AD8 and GPCOGi have fewer items than IQCODE and are arguably less time-consuming in a traditional consultation. However, with the increasing use of e-consultations, this may be less of a consideration as informant questionnaires could be sent electronically, or posted, prior to an appointment.

If an informant questionnaire does not suggest impaired cognition, this could guide the consultation to consider alternative causes for the presentation such as hearing or visual impairment or mood disorders. Indeed, one option in people over 70 years, who do not report otherwise seriously distressing symptoms, would be to use a normal IQCODE, AD8 or GPCOGi to avoid prolonged and burdensome diagnostic pathways for patients and carers, recognising that if patients or their family desired further tests then clinicians could (perhaps should) offer these. Future research should examine how informants rating of cognition might change over time and whether this differs by type of informant.

Informants provide invaluable information in helping assess the clinical diagnosis of dementia. We find little empirical evidence that the type of informant is important in ruling out a diagnosis but may differ if trying to rule in a diagnosis.

## Data Availability

The datasets used and analysed during the current study are available from the corresponding author on reasonable request.

## Abbreviations

ACE-III: Addenbrooke’s Cognitive Examination III
AD8: Ascertain Dementia 8
AUROC: Area under receiver operator curve
BADL: Bristol Activities of Daily Living
BASDEC: Brief Assessment Schedule Depression Cards
BNSSG: Bristol, North Somerset and South Gloucestershire
CIND: Cognitive impairment not dementia
DSM: Diagnostic and Statistical Manual of Mental Disorders
GP: General Practitioner/Practice
GPCOG: General Practitioner Assessment of Cognition
GPCOGi: informant component of General Practitioner Assessment of Cognition
ICD: International Classification of Disease 10th Revision
IQCODE: Informant Questionnaire on Cognitive Decline in the Elderly
NCD: Neurocognitive disorder
NICE: National Institute for Health and Care Excellence
QADAS: Quality Assessment of Diagnostic Accuracy Studies
